# Spinal Cord Stimulation in Failed Back Surgery Syndrome: Effects on Posture and Gait—A Preliminary 3D Biomechanical Study

**DOI:** 10.1155/2017/3059891

**Published:** 2017-09-25

**Authors:** L. Brugliera, A. De Luca, S. Corna, M. Bertolotto, G. A. Checchia, M. Cioni, P. Capodaglio, C. Lentino

**Affiliations:** ^1^Istituto Auxologico Italiano, Unità di Riabilitazione Osteoarticolare, Ospedale S Giuseppe, Strada Cadorna 90, Piancavallo, Italy; ^2^Laboratorio Analisi del Movimento Ospedale Santa Corona di Pietra Ligure, Viale 25 Aprile 38, 17027 Pietra Ligure, Italy; ^3^Centro Terapia del Dolore e Cure Palliative, Dipartimento di Riabilitazione, Ospedale Santa Corona di Pietra Ligure, Viale 25 Aprile 38, 17027 Pietra Ligure, Italy; ^4^Struttura Complessa Recupero e Rieducazione Funzionale, Ospedale Santa Corona di Pietra Ligure, Viale 25 Aprile 38, 17027 Pietra Ligure, Italy; ^5^Scuola di Specializzazione in Medicina Fisica e Riabilitativa, Dipartimento di Scienze Biomediche e Biotecnologiche, Università degli Studi di Catania, Piazza Università 2, 95131 Catania, Italy

## Abstract

We studied 8 patients with spinal cord stimulation (SCS) devices which had been previously implanted to treat neuropathic chronic pain secondary to Failed Back Surgery Syndrome. The aim of our study was to investigate the effects of SCS on posture and gait by means of clinical scales (Short Form Health Survey-36, Visual Analogue Scale for pain, and Hamilton Depression Rating Scale) and instrumented evaluation with 3D Gait Analysis using a stereophotogrammetric system. The latter was performed with the SCS device turned both OFF and ON. We recorded gait and posture using the Davis protocol and also trunk movement during flexion-extension on the sagittal plane, lateral bending on the frontal plane, and rotation on the transversal plane. During and 30 minutes after the stimulation, not only the clinical scales but also spatial-temporal gait parameters and trunk movements improved significantly. Improvement was not shown under stimulation-OFF conditions. Our preliminary data suggest that SCS has the potential to improve posture and gait and to provide a window of pain-free opportunity to optimize rehabilitation interventions.

## 1. Introduction

Chronic pain of moderate to severe intensity occurs in 19% of adult Europeans, seriously affecting the quality of their social and working lives [1, 2]. The prevalence of neuropathic chronic pain is estimated within 0.9 and 8% [3, 4]. Chronic pain has direct consequence on the quality of daily living and also a considerable social cost either for the national health services or for the employers [2, 5]. Failed Back Surgery Syndrome (FBSS) has been defined as “persistent or recurrent pain in the back/neck or limbs despite surgery or treatment thought likely to relieve pain.” After determining the cause of FBSS, a multidisciplinary approach is preferred, including pharmacologic management of pain, physical therapy, and behavioural intervention, as well as therapeutic procedures such as injections, radiofrequency ablation, lysis of adhesions, spinal cord stimulation, and surgical revisions. Physical therapy and medication management are the cornerstone of first-line management of FBSS. When this combined approach is not effective, neuromodulation therapies, in particular spinal cord stimulation (SCS), have to be considered. Spinal cord stimulation has been established as a cost-effective treatment for patients with neuropathic back and leg pain [6, 7]. The PROCESS study in 2007 involved 100 patients with FBSS and looked at the effects of adding SCS to usual care in comparison with usual care alone. The study demonstrated better results in terms of pain scores, quality of life, functional capacity, and patient satisfaction with the addition of SCS. More recently in the PRECISE Study, Zucco et al. [7] performed an observational, multicenter, longitudinal ambispective study on 80 patients with FBSS with predominant leg pain refractory to conventional medical treatment and followed them for up to 24 months after SCS. Although total societal costs increased after SCS placement, the authors concluded that SCS implantation would be cost-effective in 80%–85% when adjusting for quality-adjusted life years. This study underscores the continued costs of untreated FBSS on society as a whole, including loss of productivity, costs associated with disability, emergency room visits, imaging costs, and costs of medications and hospitalizations. A recent Cochrane review [8] reported a moderate level of evidence of the effectiveness of SCS in FBSS patient.

Beneficial effects of SCS on balance and risk of fall have been reported in a study on 11 subjects [9]. A case report in chronic neuropathic pain secondary to FBSS undergoing SCS showed an improvement in lower limb muscle strength and in the motor performance during gait and a reduction in claudicatio independent from the analgesic effect [10]. The author suggested that the anti-ischemic effect of SCS, which induces an increased blood flow and oxygenation of the stimulated area, may have accounted for the results.

A biomechanical study in 2005 [10] addressed the short- (1–3 months), medium- (6 months), and long-term (1 year) effects on posture and gait of SCS on untreatable chronic pain secondary to polyneuropathy, spinal stenosis, and complex regional pain syndrome. The study was conducted using 3 cinematic sensors applied to the trunk and lower limbs: 1 biaxial accelerometer to the trunk in order to measure acceleration of the trunk in the vertical and frontal planes, 1 gyroscope and accelerometer on the thigh to measure angular velocity in the sagittal plane, and 1 gyroscope on the calf to measure angular velocity in the sagittal plane. The data acquired for a 5-day period showed a significant improvement in gait (stride length, velocity, and distance walked) and physical activity levels over time. The authors concluded that SCS was effective not only in pain relief but also in improving patients' gait and physical activity levels. The gold standard in human movement studies is represented by 3D Gait Analysis, whose data yield higher specificity and sensibility as compared to the aforementioned investigation methods. The hypothesis of our research was that SCS may have a beneficial effect on posture and gait of FBSS patients.

The aim of our study was therefore to verify by means of 3D Gait Analysis whether SCS is able to improve posture and gait in FBSS patients.

## 2. Methods

### 2.1. Subjects

We studied 8 patients with SCS devices which had been implanted to treat neuropathic chronic pain secondary to FBSS. The patients were 4 males and 4 females, mean age 65.3 ± SD years. They had been previously referred for continued pain after spinal surgery to the Pain and Palliative Care Clinic of the Santa Corona Hospital in Pietra Ligure, Italy, for SCS implant. At the time of the implant, patients had been complaining of recurring or persistent leg pain, greater than back pain, despite one or more anatomically successful back surgeries for the same original pain.

The year of implant of the SCS device ranged from 2010 to 2014. Four different models of SCS device had been implanted in our 8 experimental patients. The patients' characteristics, the SCS devices and their year of implant, and the scores of the clinical scales used in the study are described in Table 1.

We studied those patients in the Movement Analysis Research Laboratory of the same hospital. The study has been performed in accordance with the Declaration of Helsinki. All subjects provided informed consent to the study approved by the Ethics Committee of the Santa Corona Hospital.

### 2.2. Evaluation

Subjects were evaluated with both clinical scales and an instrumented-quantitative evaluation in order to evaluate the effects of SCS in terms of perceived physical functioning, pain, health status, and movement.

### 2.3. Clinical Evaluation

Before (T0) and after (T1) the stimulation, all of the subjects were asked to fill the Short Form Health Survey-36 (SF-36) (Ware and Sherbourne, 1992 [11]), rate their perception of pain on the Visual Analogue Scales (VAS) [12], and provide a score on the Hamilton Depression Rating Scale (HAM-D) [13].

The SF-36 is a 36-item, patient-reported survey of patient's health. SF-36 includes one multi-item scale measuring each of 8 health concepts: (1) physical functioning; (2) role limitations because of physical health problems; (3) bodily pain; (4) social functioning; (5) general mental health (psychological distress and psychological well-being); (6) role limitations because of emotional problems; (7) vitality (energy/fatigue); and (8) general health perceptions. The 8 scaled scores are the weighted sums of the questions in their section. Each scale is directly transformed into a 0–100 scale on the assumption that each question carries equal weight. Lower scores evidence more disability.

The Visual Analogue Scale (VAS) consists of a 10 cm straight line with the endpoints defining extreme limits such as “no pain at all” and “pain as bad as it could be.” The patient is asked to mark his pain level on the line between the two endpoints. The distance between “no pain at all” and the mark then defines the subject's pain.

The HAM-D is a multi-item questionnaire used to provide an indication of depression and as a guide to evaluate recovery. The questionnaire is designed for adults and is used to rate the severity of their depression by probing mood, feelings of guilt, suicide ideation, insomnia, agitation or retardation, anxiety, weight loss, and somatic symptoms. Each item on the questionnaire is scored on a 3- or 5-point scale, depending on the item. A score of 0–7 is considered to be normal; scores of 8–17 indicate a mild depression, scores of 18–24 a moderate depression, and scores > 25 a severe depression.

### 2.4. Instrumented Evaluation

Instrumented evaluation was performed in a morning section under two different conditions: with the SCS device turned off (OFF) and on (ON).

All subjects were asked to switch off the device 12 hours before the evaluation. In the morning, they were first evaluated with the SCS device turned off (OFF). Then, the device was switched on (ON) and, after 30 minutes of SCS, they were evaluated again. This lapse of time was chosen for the patients to fully perceive the effects of the stimulation (paresthesia).

Instrumented movement analysis was performed with a stereophotogrammetric system (SMART DX, BTS Bioengineering, Milan, Italy). The system is composed of eight infrared cameras (SMART DX 5000, BTS Bioengineering) and four force platforms (P6000, BTS Bioengineering).

#### 2.4.1. Gait and Posture Analysis

We recorded the position of 22 reflective markers positioned on the patient body according to the Davis protocol [14] for 3D gait and posture analysis: spinal process of sacrum, spinal process of C7, acromion (both sides), anterior superior iliac spine, greater trochanter, lateral epicondyle of the femur, fibula head, lateral malleolus, and fifth metatarsal phalangeal joint of the foot. In both legs, two markers were placed in each leg on bars 5 cm long, at the middle point between the greater trochanter and the lateral epicondyle of the femur, and a middle point between the fibula head and the lateral malleolus.

After measuring anthropometric parameters (weight, height, hip height, hip width, knee and ankle width, and leg length) subjects were first asked to stand still on a force platform for 5 seconds; then, they were asked to walk, at their preferred speed on a 9-meter walkway at least five times to obtain a minimum of three trials complete of kinematic and kinetic data.

#### 2.4.2. Trunk Motion 3D Analysis

Trunk kinematics were assessed under three different movement conditions: flexion-extension on the sagittal plane, lateral bending on the frontal plane, and rotation on the transversal plane.

In the flexion-extension task, subjects were asked to bend forward as much as possible from an upright position with the arms loose towards the floor and the knee extended and then to lean backwards to the maximal extension.

In the lateral bending task, subjects were asked to bend the trunk sideways in the frontal plane with the arms adducted and knee extended.

In the third task, rotation in the transversal plane to the maximal excursion was required.

**Figure 1:**
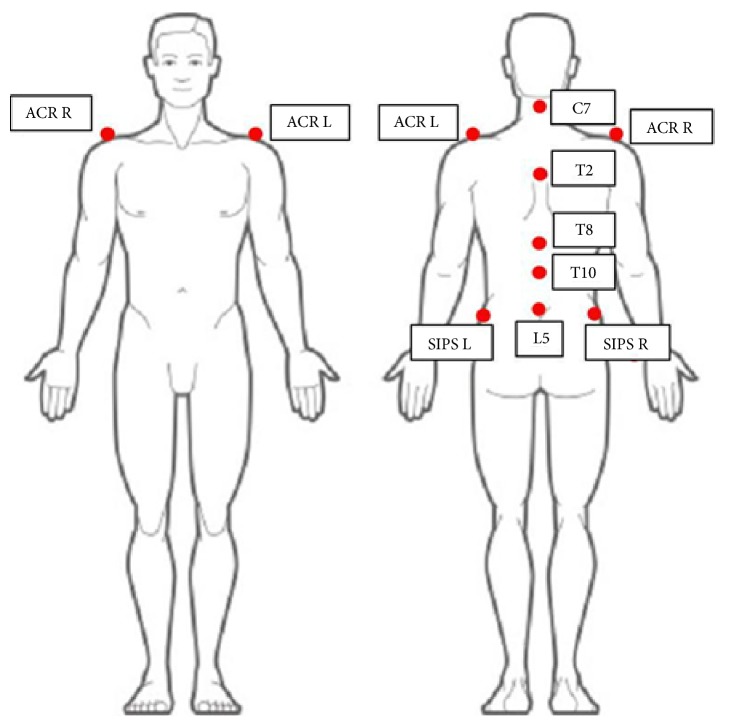
Reference points for the markers placed for the trunk motion task. SIPS, superior iliac posterior spine; ACR, acromion.

Each trial consisted of three complete movements (flexion-extension, left and right bending, and left and right rotation). Subjects performed this evaluation test with their feet placed on a force platform in order to register Center of Pressure (CoP) data during movement. We record also the position of nine markers placed on the spinal processes (C7, T2, T8, T10, and L5) and bilaterally on the acromion and the superior iliac posterior spine (Figure 1).

### 2.5. Statistical Analysis

Gait and kinematics data were analyzed by SMART Tracker and SMART Analyzer (BTS Bioengineering, Milano) and MATLAB (MathWorks, Natick, MA, US).

Student's *t*-test was performed for the comparison between stimulation OFF and ON. Data were considered significant with *p* < .05.

## 3. Results

### 3.1. Perceived Physical Functioning, Pain, and Health Status

Comparison at T0 and T1 of all self-reported scales of health status showed an improvement in performance and a reduction of negative symptoms in all subjects (Table 2, panel (A)).

### 3.2. Gait and Posture Parameters

Spatial-temporal parameters showed a general improvement. In particular, we recorded a significant improvement in velocity (*p* = 0.0032), evident in both legs and in both stance and swing phases. As for cadence, a general improvement was shown in all of the subjects, except for 2 (subjects 1 and 4), where statistically significant reduction of the cadence was recorded. A small but significant increase in step length was evident in all of the subjects during ON. Also, improvement in stride length was demonstrated. Finally, a significant improvement of the left-right symmetry of the gait cycle, due to an increase of the stance phase duration on left side during ON, was evident in all subjects. Those improvements produced an overall reduction of the stance phase and an increase of the swing phase of the gait cycle, more evident on the unaffected side (Table 2, panel (B)).

### 3.3. Force Platform Data during Gait Cycle

As for ground reaction forces (GRF), we considered both the vertical and anterior-posterior components.

### 3.4. Vertical Component

We considered the maximum peak of the GRF vertical component. Comparison between OFF and ON showed inconsistent increases in vertical force peaks; therefore no statistical significance was evident. Different strategies were observed: in 3 subjects (numbers 3, 4, and 6) the pattern was normal during OFF and did not change during ON; in 1 subject (number 2), both limbs showed an increase of both first and second peaks during ON; in 2 subjects (numbers 5 and 7) an improvement on the affected side was present during ON. During ON, no differences on the affected side were observed in 1 subject (number 1), and in another subject (number 8), only a small increase in the vertical force peak was evident.

### 3.5. Anteroposterior Component

**Figure 2:**
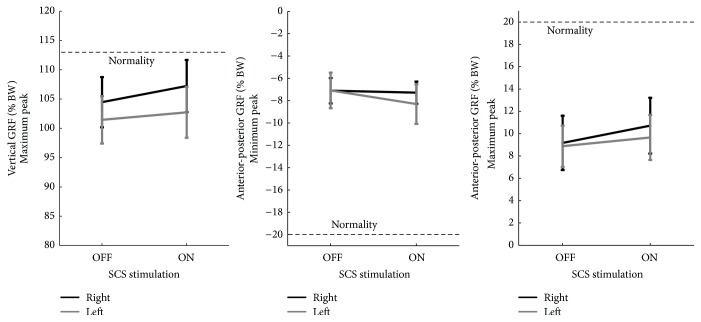
Changes in GRF peak values on both sides under ON and OFF conditions.

Mean values in both left and right limb for this component under OFF and ON conditions were calculated: +1.54 ± 0.66 SE (*p* = 0.0517) for the right limb and +0.78 ± 0.25 SE (*p* = 0.01) for the left (Figure 2).

### 3.6. 3D Analysis of Trunk

#### 3.6.1. Lateral Bending

Subjects performed in the coronal plane three lateral bending movements to the right and three to the left during the OFF and the ON phases. Amplitude and pattern of motion were observed. During ON, we observed in all of the patients a modification of the trunk strategy, resulting in a smoother task execution. In 4 patients, the smooth execution was also due to an additional movement of the trunk on the sagittal plane.

#### 3.6.2. Range of Motion (ROM)

**Figure 3:**
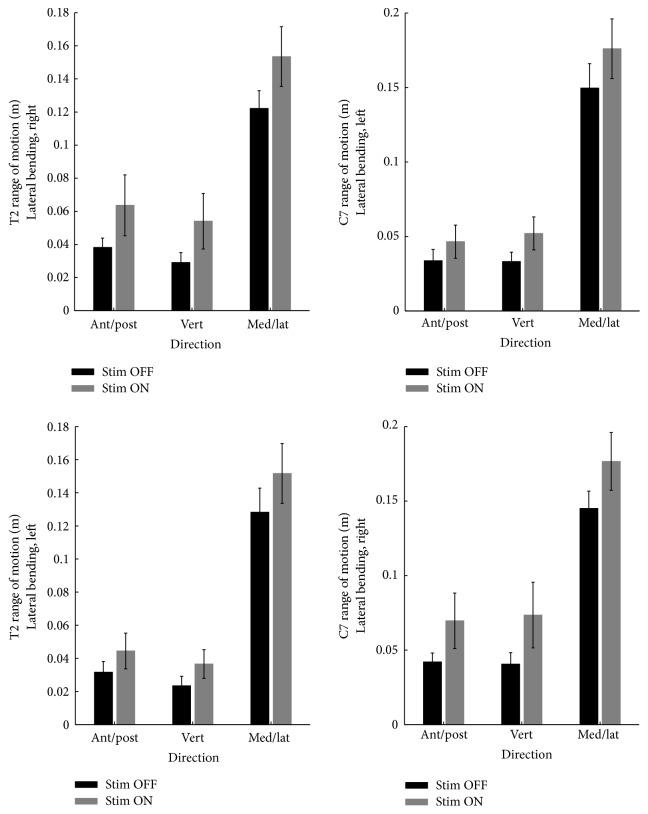
Improvements in cervical and dorsal spine ROM in lateral bending on both sides during ON.

As shown in Figure 3, under the ON condition, markers placed at C7 and T2 during the left-to-right bending showed an increase in ROM in all of the three axes; such increase was statistically significant in the coronal plane.

#### 3.6.3. Bending to the Right Side

In 4 subjects (numbers 2, 4, 6, and 8), an increased ROM in the coronal plane corresponded to an increase in the vertical axis but also to a higher forward shift of the trunk. In 2 subjects (numbers 5 and 7), an increased ROM in both vertical and coronal components and a reduction of the forward bending were evident. One subject (number 3) showed an increased ROM only in the coronal plane and in another subject (number 1), no difference in ROM was evident between OFF and ON.

#### 3.6.4. Bending to the Left Side

Three subjects (numbers 2, 4, and 8) had the same pattern as in the contralateral bending. In three subjects (numbers 2, 5, and 7), an increase in the vertical and lateral ROM and a decrease in forward bending were evident. One subject (number 3) showed an increase in lateral and forward bending components but a decrease in the vertical one. One subject (number 6) showed a reduction in the vertical one.

Other indexes, such as path length, duration, distance, smoothness index jerk (not shown in the paper), and speed, were also analyzed. However, speed was the best index to demonstrate the improvement of performance during the ON phase (Table 2, panel (C)).

## 4. Discussion

Patients chronically exposed to pain adopt compensatory strategies in order to avoid painful movements. This compensation can result in abnormalities of gait and posture. We felt therefore that investigating quantitative changes in gait and posture in FBSS patients after SCS would provide some initial evidence on the functional effectiveness of the treatment. The relationship between functioning of the spine and pain has been extensively addressed; however, we are aware that research on its correlation is still generally equivocal and considerations about the relationship between pain and functioning were beyond the scopes of our preliminary study.

Gait Analysis, the gold standard for quantification of the cinematics of human movement, was used in this study for that purpose.

Kinematics of gait and of the spine showed improvement during ON but not under the stimulation-OFF condition. During ON, improved kinetics of the lower limbs are evident: data indicate lower loading at joints level in the unaffected limb. At spinal level, compensatory strategies appear reduced but not eliminated, suggesting that neuromodulation itself may not be able to influence all of the factors involved in pain-related disuse (i.e., muscle contractures and reduced muscle strength and volume).

In our study, patients were evaluated in the morning under two different conditions: (1) stimulation OFF for the previous 12 hours, (2) 30 minutes after the stimulator had been ON. The latter condition served to investigate not only the effects on pain relief, but also the direct impact on gait and posture.

Our preliminary data indicate that stimulation positively affects patient's walking and reduces compensatory patterns, as shown by kinematic parameters. This provides some initial evidence that SCS may be useful in providing a window of pain-free opportunity to intensify rehabilitation interventions and to maximize function. This could widen the range of effective interventions of rehabilitation professionals and boost further research in the rehabilitation of complex chronic pain patients.

Data from the trunk during ON showed an increase in range of motion and smoothness of the trunk motion during the tasks. A reduced compensatory strategy of the trunk was also evident. Our results provide quantification of the compensations occurring in posture and gait in patients with chronic pain and a deeper insight into the components of gait or trunk control that improve after implant of an SCS device. A better understanding of these mechanisms can help generate tailored and more effective rehabilitation programs. For instance, reduction of ground reaction forces on the unaffected lower limb improves the symmetry of loading, thus alleviating joint overload. From the 3D analysis of the trunk motion, a smoother movement was evident. Meanwhile, the compensation strategies partially persisted, meaning that pain could not entirely account for the modifications observed in movement patterns. These observations have to be taken into account for ad hoc rehabilitation programs.

Our preliminary study yields some evident limitations: firstly, the study is performed in 8 patients only, 4 of whom complained of hypoaesthesia and 4 of disaesthesia; secondly, there is a lack of appropriate sham or concealed observation; thirdly, our results refer exclusively to the immediate effects of neuromodulation; and also, different models of SCS device had been previously implanted in our 8 subjects at different times, from 2010 to 2014, which, together with different positioning of the electrodes, depending on the type of pain, may have influenced results. However, modalities of neuromodulation delivery were consistent across the types of devices used and, technically, the devices differed only in recharging capacity and compatibility with MRI investigations. Therefore, any generalization about the clinical utility of SCS in FBBS from our data is premature and needs support from larger and also long-term studies.

## 5. Conclusion

A multilevel kinetic and cinematic 3D analysis in a small sample of patients with failed back syndrome with SCS implant has provided additional quantitative information regarding posture and gait modifications secondary to spinal cord stimulation.

## Figures and Tables

**Table 1:** The general picture at T0: patients' clinical features, model of SCS device, year of implant of the device, clinical scales (Visual Analogue Scales (VAS), Hamilton Depression Rating Scale (HAM-D), and Short Form Health Survey-36 (SF-36)), and functional scores (Medical Research Council, Timed Up and Go). HAM-D scores of 0–7 are considered to be normal; scores of 8–17 indicate a mild depression, scores of 18–24 a moderate depression, and scores > 25 a severe depression.

Age [years]	Pain localization	Type of pain	SCS device	Year of implant	VAS (0–100)	HAM-D	SF-36
79	Lower back and left lower limb	hypoesthesia	SYNERGY VERSITREL MEDTRONIC	2013	80	18	20
52	Lower back and left lower limb	disesthesia	VECTRIS SURESCAN MRI MEDTRONIC	2014	90	21	5
73	Lower back and left lower limb	disesthesia	VECTRIS SURESCAN MRI MEDTRONIC	First in 2005 Second in 2014	80	13	7
51	Lower back and left lower limb	disesthesia	SINERGY VERSITREL MEDTRONIC	First in 2005 Second in 2010	100	25	10
75	Lower back and right lower limb	hypoesthesia	PRIME ADVANCED MEDTRONIC	2014	80	24	18
61	Lower back and right lower limb	disesthesia	PRIME ADVANCED MEDTRONIC	First in 2012 Second in 2014	100	43	0
55	Lower back and left lower limb	hypoesthesia	ITREL 4 MEDTRONIC	2010	90	29	10
76	Lower back and left lower limb	hypoesthesia	ITREL 4 MEDTRONIC	2013	80	15	30

**Table 2:** Panel (A) shows delta value ± standard error (SE) and *p* value (*t*-test) for Short Form Health Survey-36 (SF-36) items, Visual Analogue Scales (VAS), and Hamilton Depression Rating Scale (HAM-D) before and after implantation of SCS devices. Panel (B) shows delta value ± standard error (SE) and *p* value (*t*-test) for spatial-temporal parameters from the Davis protocol. Panel (C) shows delta value ± standard error (SE) and *p* value (*t*-test) for the 3D analysis speed index during lateral and frontal banding of the trunk.

Clinical scales			Gait parameters				3D trunk banding speed index			
*SF-36*			*Speed (m/sec)*				*Lateral (m/sec)*			
Physical functioning	39.73 ± 6.42	*p* < 0.0001	Stance phase	R	0.13 ± 0.03	*p* = 0.0032	R	Outward	0.02 ± 0.008	*p* = 0.03
Physical health	37.53 ± 6.42	*p* = 0.0557		L	0.14 ± 0.02	*p* = 0.0018		Return	0.04 ± 0.01	*p* = 0.03
Limitation										
Emotional problem	66.67 ± 14.09	*p* = 0.0021	Swing phase	R	0.12 ± 0.02	*p* = 0.0037	L	Outward	0.01 ± 0.005	*p* = 0,06
Energy/fatigue	30 ± 6.50	*p* = 0.0024		L	0.10 ± 0.04	*p* = 0.057		Return	0.03 ± 0.01	*p* = 0.02
Emotional well-being Being	35.50 ± 8.19	*p* = 0.0034	*Cadence (step/min)*		9.59 ± 3.26	*p* = 0.02	*Frontal (m/sec)*			
Social functioning	59.38 ± 5.15	*p* < 0.0001	*Step length (m)*					Forward	0.06 ± 0.03	*p* = 0.07
	58.13 ± 7.51	*p* < 0.0001	Stride	R	0.09 ± 0.02	*p* = 0.01		Backward	0.08 ± 0.02	*p* = 0.02
General health	24.45 ± 5.20	*p* = 0.0022		L	0.08 ± 0.01	*p* = 0.003				
*Health status*	49.87 ± 6.37	*p* < 0.0001		*Symmetry (% cycle)*	−2.97 ± 1.12	*p* = 0.03				
*VAS*	−51.25 ± 4.79	*p* < 0.0001								
*HAM-D*	−15.38 ± 2.26	*p* < 0.0001								

*Panel (A)*			*Panel (B)*				*Panel (C)*			

## Abbreviations

FBSS: Failed Back Surgery Syndrome
SCS: Spinal cord stimulation
3D: Three-dimension
CoP: Center of Pressure
SIPS: Posterior superior iliac spine
GRF: Ground reaction forces
ROM: Range of motion
VAS: Visual Analogue Scale
36-SF: Short Form Health Survey-36
HAM-D: Hamilton Depression Rating Scale.

## Additional Points

*Availability of Data and Material*. The datasets used and/or analyzed during the current study are available from the first author on reasonable request.
